# *Selliguea kachinensis* (Polypodiaceae), a new fern species of uncertain affinity from Northern Myanmar

**DOI:** 10.3897/phytokeys.62.8101

**Published:** 2016-03-25

**Authors:** Phyo Kay Khine, Stuart Lindsay, Christopher Fraser-Jenkins, Jürgen Kluge, Myint Kyaw, Peter Hovenkamp

**Affiliations:** 1 Faculty of Geography, Philipps University of Marburg, Deutschhausstraße 10, D-35032, Marburg, Germany; 2 Gardens by the Bay, 18 Marina Gardens Drive, Singapore 018953; 3 Student Guest House, Thamel, PO Box 5555, Kathmandu, Nepal; 4 Nature and Wildlife Conservation Division, Forest Department, Ministry of Environmental Conservation and Forestry, Office No. 39, Nay Pyi Taw, Myanmar; 5 Naturalis Biodiversity Center, PO Box 2317 2300 RA Leiden, The Netherlands

**Keywords:** New species, new combination, generic placement, conservation status, taxonomy, morphology, Arthromeris, Southeastern Himalaya

## Abstract

We describe *Selliguea kachinensis* as a new species from Northern Myanmar and discuss its generic placement in either *Selliguea* or *Arthromeris*. The conservation status is assessed as Data Deficient. In addition, we make the new combination *Selliguea erythrocarpa* (Mett. ex Kuhn) Hovenkamp, S. Linds., Fraser-Jenk.

## Introduction

During exploration of the “northern forest complex” on the eastern slope of the Myanmar-India watershed (Kachin State, Myanmar), between November 2013 and October 2014, Khine et al. and Miehe et al. collected an epiphytic fern that could be assigned to the Polypodiaceae but not be to any known species, or even easily placed in an existing genus of Polypodiaceae. After comparing it with all known species of the morphologically closest genera *Selliguea* Bory and *Arthromeris* (T. Moore) J. Sm. we have come to the conclusion that it represents a new species, but we have decided not to erect a new genus to accommodate it. The new species is here described in the genus *Selliguea*.

## Material and methods

Morphological characters were examined in the field and on herbarium specimens, and using Light (LM) and Scanning Electron microscopy (SEM). For LM, small parts of rhizome and lamina were boiled in water until they sank, and then either sectioned on a Reichert slide microtome or mounted whole without staining in glycerine jelly, and photographed using a Zeiss V20 or a Zeiss AxioImager M2 with an MRc5 digital camera and AxioVision software (Zeiss). For SEM spores were sputter-coated with 10 nm Platinum/Palladium (80/20) in a Quorum Q150TS sputter-coater, and observed with a Jeol JSM 7600F FEG-SEM. For the conservation assessment, Area of Occupancy (AOO) and Extent of Occurrence (EOO) were estimated using GeoCAT (Bachman et al. 2011), with default settings for grid size. The specimens collected by Khine et al. and Miehe et al. are kept at the Faculty of Geography, Philipps University of Marburg, with duplicates distributed to L, RAF and SING (abbreviations according to Thiers, continuously updated).

## Results

### Selliguea kachinensis

Taxon classificationPlantaePolypodialesPolypodiaceae

Hovenkamp, S. Linds., Fraser-Jenk. sp. nov.

urn:lsid:ipni.org:names:77153918-1

#### Type.

Myanmar, Kachin State, Hponyin Razi, Quercus-Magnolia-Araliaceae forest, epiphyte. 27.601421°N, 96.988873°E, 1715 m, G. Miehe, P.K. Khine [“Kine”], L. Shein, M. Kyaw, P. Ma, S. Lan Wan 13-094-159, 19 Nov. 2013 (holotype: L; isotype: SING).

#### Epiphytic.

Rhizome long-creeping, branched, 3.2–4.5 mm diam. when dry (c. 6 mm diam. after soaking in boiling water), black and shiny when dry with a glaucous waxy layer, the younger parts densely covered with scales, irregularly rooting from the ventral side, in cross-section with scattered sclerification in the peripheral, epidermal and subepidermal region; phyllopodia c. 2 cm distant, c. 1–3 mm high. Scales deciduous, mostly absent from older parts of the rhizome, basifixed and slightly to strongly auriculate, or pseudopeltate, or sometimes fully peltate, c. 0.5 × 2.0–3.5(–4.0) mm, gradually narrowed from the base to a long narrow acumen, brown or blackish near the attachment, central region brown and thick, the margin and acumen thinner and lighter, margin irregularly dentate, more strongly so towards the base. Fronds pendent, simple, monomorphic, stipitate, all parts densely hairy with multicellular, uniseriate, soft hairs to 1.5(–1.7) mm long, the longest hairs inserted on midrib and veins; sparse, long narrow pale strongly toothed scales present among the hairs on the abaxial midrib (particularly towards the base of the lamina), stipe 0.8–5.0 cm long, c. 1 mm thick; lamina 18–58 × 3.3–7.2 cm, oblong – narrowly elliptic, the basal 1-6 cm often narrowed, base truncate to cordate, apex acuminate, texture thin-herbaceous, glaucous when fresh, translucent when dry, margin very narrowly hyaline, without notches. Venation anastomosing, primary veins straight or slightly curved, at 60–90 degrees to the midrib, secondary veins hardly distinct, delimiting c. 5–6 rows of rectangular areoles with anastomosing tertiary veins and free veins in all directions, ending in hydathodes. Sori in a single row between each pair of primary veins, usually one per areole, but sometimes absent from the first one or two areolae closest to the midrib and occasionally two in areolae closest to the margin, c. 2 mm in diameter when ripe. Sporangia long-stalked, capsules c. 0.2 mm long, bearing 2–6 uniseriate, c. 0.4–0.8 mm long hairs, annulus with 14–16 indurated cells. Spores 29–46 × 25–34 µm in lateral view, perispore with a 0.1–0.3 µm thick, finely colliculate basal layer, rather densely set with narrow, fragile spines, spines c. 2 µm long by 0.5 µm thick at the base, somewhat narrowed to a blunt apex, apparently easily breaking off at the base leaving a low round scar.

#### Additional specimens seen.

Myanmar, Kachin State. Hponyin Razi: G. Miehe, P.K. Khine [“Kine”], L. Shein, M. Kyaw, P. Ma, S. Lan Wan 13-096-034, 23 Nov. 2013, above 1300 m, road site (L); G. Miehe, P.K. Khine [“Kine”], L. Shein, M. Kyaw, P. Ma, S. Lan Wan 13-131-013, 11 Nov. 2013, 1600 m, road site (SING).

Hponkan Razi: P.K. Khine [“Kine”], J. Kluge, A.S. Lanwan, D.R. Lanwan, P. Lanwan 14-031-020, 14 Oct. 2014, 27.548702 N, 97.032742 E, 1565 m, evergreen broadleaved (L). Above Ziadam: P.K. Khine [“Kine”], J. Kluge, A.S. Lanwan, D.R. Lanwan, P. Lanwan 14-047-022, 21 Oct. 2014, 27.585061°N, 97.104085°E, 1448 m, evergreen broadleaved (L, SING). Pisa District: Wang Jun & Zhou Lian Xuan 5431, 18 Apr. 2009, Pangjia (26°31.589'N, 98°18.473'E, 717 m) to Wuru (26°31.589'N, 98°18.473'E), in forest on a tree (CDBI). Ridan: Xia Nianhe, Deng Yunfei, Zhou Wei & Wu Linfang 1519, 20 Mar. 2009, around the Waqkure village, c. 2 miles from Ridan, E. side of Namai Kha river, 27°11.876'N, 98°15.165'E, 1400 m., in forest (CDBI).

#### Etymology.

The name derives from Kachin State, where the species is found.

#### Ecology.

Based on the specimens collected in the “northern forest complex”, *Selliguea kachinensis* grows on heavily moss-covered trees in primary evergreen broadleaved forest (dominated by *Fagaceae*, *Lauraceae*, *Araliaceae*, and *Magnoliaceae*) between 1300 m and 1715 m. It was found occasionally on trunks at 4 m from the ground (Figure 3), more frequently in the moss cover of trunks and thicker branches above 8 m and in the tree crowns, but is absent in the outer canopy. It is locally abundant together with *Drynaria propinqua* (Wall. ex Mett.) J.Sm. (Figure 4). It was not found growing on steep rock cliffs or open banks along trails. During our visits in November 2013, and October 2014 we did not observe any wilting of the fronds (in contrast to *Oleandra neriiformis* Cav. and *Oleandra wallichii* C.Presl which are lithophytes/epiphytes with a somewhat similar habit) and so could not assess whether it is deciduous or evergreen, but the herbaceous texture suggests that it is deciduous.

**Figure 1.**
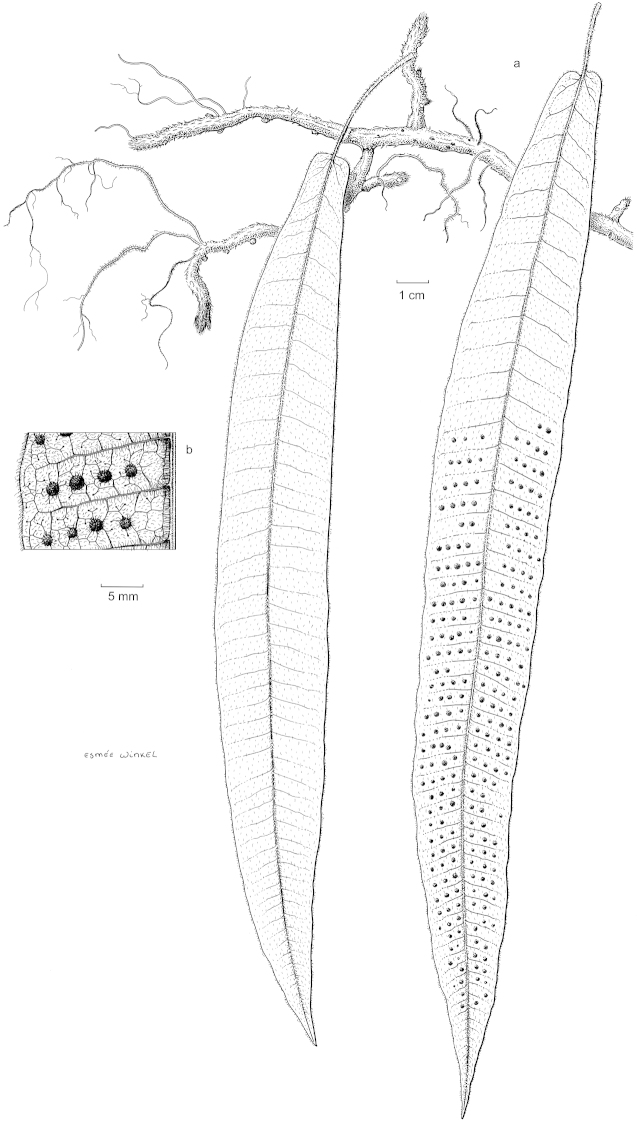
*Selliguea kachinensis*. **A** habit **B** detail showing venation pattern. After Kine *et al*. 14-047-022 (L). Drawing by Esmée Winkel.

**Figure 2.**
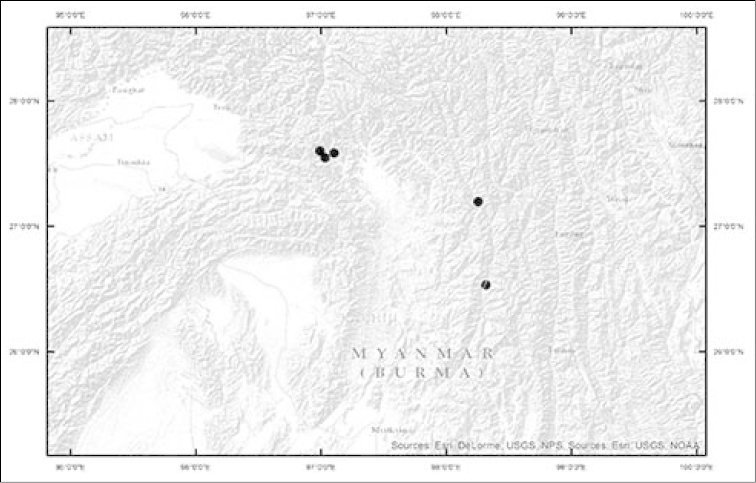
*Selliguea kachinensis* – Distribution.

**Figure 3.**
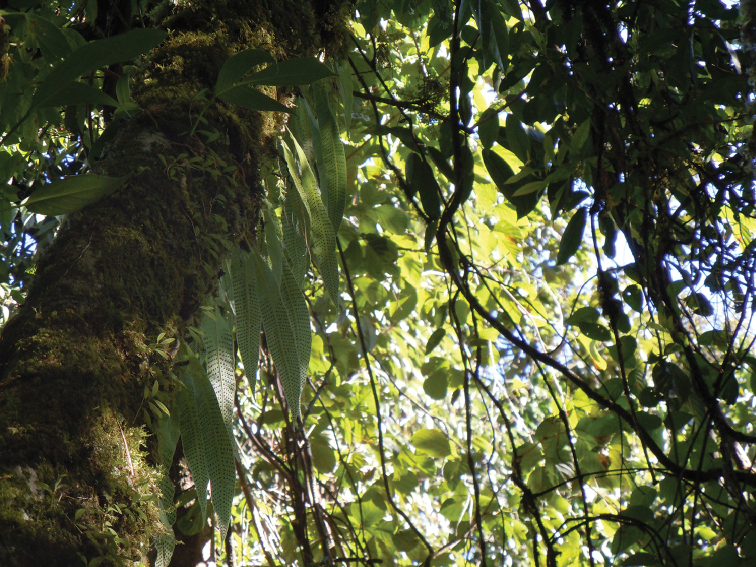
Habitat of *Selliguea kachinensis*: a thick bryophyte covered trunk in Hponyin Razi at about 1,700 m (photograph by P. K. Khine).

**Figure 4.**
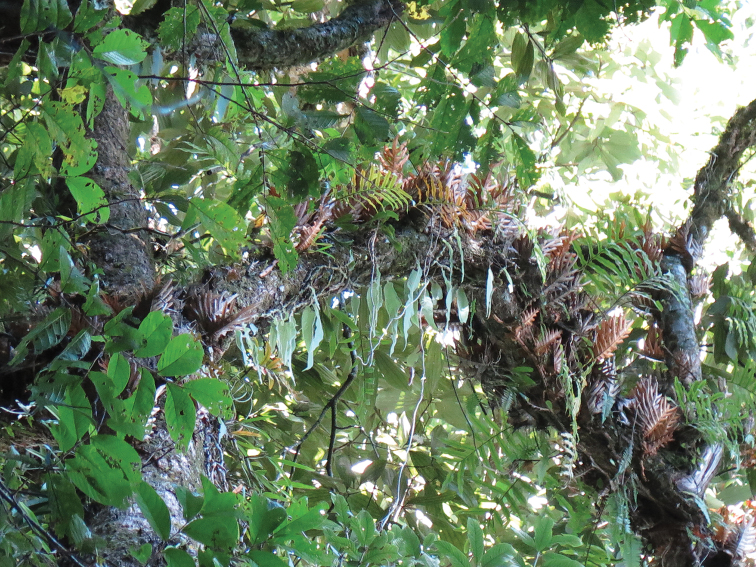
Habitat of *Selliguea kachinensis*: a thick branch covered with other Polypodiaceae such as *Drynaria propinqua*, and Orchidaceae in Hponkan Razi at 1,600 m (photograph by P. K. Khine).

#### Climate.

The climate station nearest to the collection sites is Putao (450 m a.s.l) in an intramontane basin 50 km to the southeast. It records approximately 4000 mm rainfall between May and October with a pronounced dry season from November to January. We expect that the annual rainfall at altitudes between 1400 and 1800 m a.s.l. exceeds 5000 mm plus an unknown amount of fog precipitation from clouds shrouding the mountains between April and November.

#### Distribution, conservation and threats.

*Selliguea kachinensis* is currently known from five locations, all in the north of Kachin State, Myanmar. On the basis of these occurrences, the Extent of Occurrence is 4738 km2, while the known Area of Occupancy is 20 km2. However, as exploration of the area has been very fragmentary, we have little information on the actual occurrence of this species, which could well be more widely distributed along the rim of the Mali Kha / Irawaddy River basin. To date, forests where *Selliguea kachinensis* is found are among the least disturbed submontane evergreen broadleaved forests of the Southeastern Himalaya. Drastically increased population could extend swidden farming and might lead to a reduction of the population, but we have no information on any concrete and current threats to the habitat of the species. Accordingly, we propose a status of Data Deficient (IUCN 2014).

#### Discussion: generic placement.

**Figure 5.**
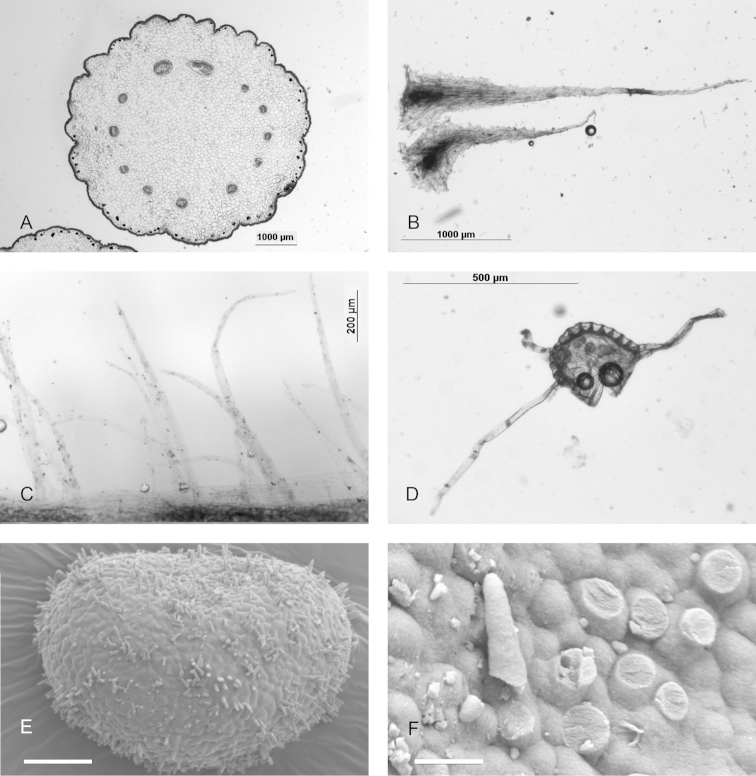
*Selliguea kachinensis*. **A** cross section of rhizome **B** rhizome scales **C** lamina margin **D** sporangium **E** spore **F** detail of spore, scale bar. All from the holotype, Miehe *et al*. 13-094-159 (L). Scale bar: 10 µm (**A–E**); 1 µm (**F**).

*Selliguea kachinensis* does not fit easily into the genus *Selliguea*, which contains mostly species with a more coriaceous texture and a distinctly cartilaginous, often notched, margin (although a thin-herbaceous texture is notably present in *Selliguea pui* Hovenkamp). An alternative position would be in the related genus *Arthromeris*. This would agree with the rather distinctive, glabrescent rhizome, which is similar to that of *Arthromeris lehmanii* (Mett.) Ching or *Arthromeris tomentosa* W.M.Chu, and with a number of other characters (Table 1) but it would seriously weaken the diagnostic value of that genus, as all species so far placed in *Arthromeris* have imparipinnate fronds with articulate pinnae (Lu and Hovenkamp 2013; Tagawa and Iwatsuki 1989). There are several distinctive characters in *Selliguea kachinensis* that argue against placement in either of these genera, and for the erection of a new genus. The often somewhat lyrate base of the lamina of *Selliguea kachinensis* is distinctive and not encountered in any other species of either *Selliguea* or *Arthromeris*. The rhizome of *Selliguea kachinensis* is also distinct in that the cross-section shows sclerification only in the peripheral, subepidermal region (Figure 5a). Sclerification occurs frequently in *Selliguea*, rarely in *Arthromeris*, but in both cases takes the form of sclerified strands in the central part of the rhizome, or a continuous, sclerified band well below the epidermis. The rhizome scales (Figure 5b) do not show any distinctive characters. A dense indument of long hairs similar to the indument of *Selliguea kachinensis* (Figure 5c, d) occurs in some species of *Arthromeris*, but in *Selliguea*, *Selliguea trisecta* (Baker) Fraser-Jenk. and *Selliguea erythrocarpa* (Mett. ex Kuhn) Hovenkamp, S. Linds., Fraser-Jenk. *comb. nov.* (basionym: *Polypodium erythrocarpum* Mett. ex Kuhn, Linnaea 36: 135. 1869) are also hairy, while *Selliguea chrysotricha* (C.Chr.) Fraser-Jenk. also has hairs (albeit short and stiff ones) on the capsules of the sporangia. The spore ornamentation (Figure 5e, f) is matched in *Selliguea* by e.g. *Selliguea quasidivaricata* (Hayata) H.Ohashi & K.Ohashi and *Selliguea yakushimensis* (Makino) Fraser-Jenk. and in *Arthromeris* by e.g. *Arthromeris tenuicauda* (Hook.) Ching and *Arthromeris lehmannii* (Mett.) Ching (Tryon and Lugardon 1991). Thus, there are arguments both for and against placement in *Selliguea* or in *Arthromeris* and there are arguments in favour of erecting a new genus. We have decided not to do the latter, as the generic taxonomy of the Selligueoid ferns is at the moment unsettled, has been burdened already by the erection of numerous small genera (Hovenkamp 1998), and it is beginning to become clear that the best option to avoid paraphyletic groups may be to accept a large genus *Selliguea* (He et al. in prep.). As alternative to a monotypic genus, we prefer a placement in the genus *Selliguea* over one in *Arthromeris* in anticipation of a generic reorganization along these lines.

**Table 1.** Comparison of morphological characteristics of *Selliguea kachinensis* with *Selliguea* and *Arthromeris*.

	*Selliguea*	*Selliguea kachinensis*	*Arthromeris*
Rhizome with scattered sclerification	Absent or central	Peripheral	Absent or central
Rhizome with continuous sclerified band	Often	No	No
Lamina shape	Simple to pinnate	Simple	Pinnate, pinnae articulate
Lamina texture	Mostly coriaceous	Thin-herbaceous	Thin-herbaceous to herbaceous
Lamina indument	Glabrous to short-hairy	Soft hairy	Glabrous to densely soft-hairy
Lamina margin	Mostly cartilaginous, often notched	Not differentiated	Often distinctly flat-cartilaginous, not notched
Sporangial indument	Rarely present, short stiff hairs	Soft long hairs	Absent

## Supplementary Material

XML Treatment for Selliguea kachinensis
